# Well-designed manufacturing work improves some cognitive abilities in individuals with cognitive impairments

**DOI:** 10.3389/fresc.2024.1377133

**Published:** 2024-05-14

**Authors:** Pamela Banta Lavenex, Marie-Laure Blandin, Caroline Gaborieau, Pierre Lavenex

**Affiliations:** ^1^ Faculty of Psychology, UniDistance Suisse, Brig, Switzerland; ^2^ AMIPI Foundation—Bernard Vendre, Cholet, France; ^3^ Laboratory of Brain and Cognitive Development, Institute of Psychology, University of Lausanne, Lausanne, Switzerland

**Keywords:** well-designed work, cognitive training, cognitive enhancement, fluid intelligence, intellectual disability

## Abstract

**Introduction:**

Employment is recognized as a fundamental human right, which correlates with better physical and mental health. Importantly, well-designed work, which considers the physical, social, and psychological impacts of work, can serve to enhance the cognitive abilities of workers. Although often overlooked, work for individuals with disabilities, including cognitive impairments, is equally important for their physical and mental well-being. What has not been established, however, is whether well-designed work can also enhance the cognitive abilities of individuals with cognitive impairments.

**Methods:**

Using a longitudinal study design, we investigated the impact of well-designed work on the cognitive abilities of 60 participants (operators) at the AMIPI Foundation factories, which employ individuals with cognitive impairments to produce electrical cables and harnesses for the automobile industry. The same operators were assessed at three different time points: upon hiring (*n* = 60), and after working in the factory for 1 year (*n* = 41, since 19 left the factory) and 2 years (*n* = 28, since 13 more left the factory). We used five cognitive tests evaluating: (1) finger and manual dexterity, bimanual dexterity, and procedural memory using the Purdue Pegboard; (2) sustained and selective attention using the Symbol Cancellation Task; (3) short- and long-term declarative verbal memory and long-term verbal recognition memory using Rey's Audio-Verbal Learning Test; (4) short- and long-term visual recognition memory using the Continuous Visual Memory Test; and (5) abstract reasoning using Raven's Standard Progressive Matrices.

**Results:**

We observed improvements in procedural memory, sustained and selective attention, and short- and long-term visual recognition memory after working in the factory for 1 or 2 years. We did not observe improvements in finger or manual dexterity or bimanual dexterity, nor short- or long-term declarative verbal memory or verbal recognition memory, nor abstract reasoning.

**Discussion:**

We conclude that, in addition to improving physical and mental well-being, well-designed manufacturing work can serve as a training intervention improving some types of cognitive functioning in individuals with cognitive impairments.

## 1 Introduction

According to Article XXIII of the United Nations Declaration of Human Rights, employment is recognized as a fundamental human right, and as such individuals should be guaranteed the right to work, to freely choose their employment, to dignified working conditions, and to protection from unemployment (1, 2). Employment plays a fundamental role in physical and mental health (3–9). Employment has been identified as a social determinant of health, a non-medical factor that influences health outcomes at both the individual and group levels (10). Gainful employment is associated with better physical health outcomes (5, 11), and better psychological well-being (8, 9). In contrast, unemployment and underemployment are associated with worse physical health outcomes (4, 5), and poorer mental health including higher rates of depression and psychological distress (6, 12).

Employment plays an equally important role in the physical and mental health, and quality of life of individuals with mental illness or cognitive impairments (1, 13–18). The term cognitive impairment describes disabilities that impair one or more aspects of cognitive functioning, such as learning, memory, language processing, attention, planning, reasoning, or decision-making (19, 20). Cognitive impairments can result from a wide range of developmental or acquired conditions, including intellectual, developmental or learning disabilities, injury, mental illness, and neurodegenerative disorders (19, 20). Excluding individuals with disabilities from equitable employment leads to a range of social injustices including disempowerment, socio-political vulnerability, and poverty (21). Accordingly, Article XXVII of the United Nations Convention on the Rights of Persons with Disabilities extends the above-mentioned rights of employment to individuals with long-term physical, mental, cognitive or sensory impairments (22).

A large body of literature has provided evidence for the positive association between one's participation in meaningful activities and cognitive performance in neurotypical individuals. Whereas the term “occupation” was historically associated with work, the occupational perspective defines it as a way of looking at or thinking about “human doing” (23). Today, the concept of “occupation” can be understood to refer to the meaningful activities that humans engage in to occupy our time (23–25). It is important, then, that various activities in which humans engage to occupy their time have been shown to correlate positively with cognitive functioning, including reading (26), singing or playing a musical instrument (27), social activities (28, 29), and leisure activities (30). Moreover, some studies suggest even greater effects on cognitive functioning when individuals practice several different activities rather than any single activity (31, 32). Some research further suggests that the relationship between occupation and improved cognitive functioning is mediated by intellectual engagement (33). For example, in their longitudinal study of a large cohort of Scottish sexagenarians spanning more than five decades, Staff and colleagues (33) found that early and sustained intellectual engagement across the lifespan was correlated with better cognitive functioning later in life. However, although self-reported retrospective levels of intellectual engagement were associated with cognitive ability in both childhood and late adulthood, intellectual engagement did not influence the rate of age-related decline. The authors therefore postulated that early and sustained intellectual engagement, especially problem-solving, contributes to an enhancement in cognitive functioning (33), or the creation of what is known as “cognitive reserve” (34, 35). Greater cognitive reserve is presumed to provide intellectually engaged individuals with an initially higher level of cognitive ability prior to the onset of inevitable age-related cognitive decline (33).

It is particularly concerning, then, that individuals with cognitive impairments often lead less occupied lives. Children and adults with cognitive impairments are often less physically active (36–40). Since research is continually revealing the critical relationship between physical activity, brain health, and cognitive function (41–45), reduced physical activity in children and adults with cognitive impairments may have a particularly detrimental impact on cognitive performance (46–48). Individuals with cognitive impairments also participate less in intellectually engaging activities as compared to typical individuals in several ways: First, the educational experience of individuals with cognitive impairments is usually truncated as compared to their peers (49, 50), depriving them of several years of crucial and highly stimulating intellectual activity; Second, individuals with cognitive impairments are more socially isolated than typically developed individuals (51–56), depriving them of critical social stimulation that is beneficial to cognitive functioning (29, 57, 58); Third, due to their intellectual limitations, social isolation, and inability to independently translate motivation into action, such as independently identifying and joining an inclusive activity that they are physically able to regularly attend, individuals with cognitive impairments are less likely to engage in intellectually engaging activities such as book clubs, musical groups or other leisure activities that have been associated with improved cognitive performance (59). As a consequence, from mid-adolescence through adulthood many individuals with cognitive impairments live less occupied and less intellectually engaged lives.

Given that intellectual engagement is positively associated with cognitive functioning and performance, and that intellectual engagement can be an incidental or intentional consequence of occupations such as leisure activity and work, researchers have been interested in how work may contribute to building cognitive reserve and preserving cognitive performance during aging in neurotypical individuals (33, 60–62). Organizations of all types are concerned with the long-term cognitive performance of their workers, as it directly subserves their ability to acquire and maintain the knowledge and skills needed to perform their jobs (63, 64). The field of work design is concerned with understanding how work-related tasks, activities, relationships and responsibilities can be optimized to improve worker intellectual performance and physical and mental well-being (65). Researchers have identified five key features of work design that can influence employee performance over the long-term: job complexity; worker autonomy; performance feedback; social aspects of work such as interdependence and social support; and psychosocial demands which pertain to the physiological or psychological costs associated with jobs that require sustained physical or mental effort (61). In contrast to work environments that do not consider the employees' physical, mental or intellectual well-being, well-designed work and work environments take into consideration these five key factors (66). Over the short- to medium-term, from a few months to a few years, well-designed work can offer employees the opportunity to apply their knowledge and use their cognitive abilities to enhance their problem-solving abilities (fluid intelligence), to accelerate knowledge acquisition (crystallized intelligence), and to engage in learning-oriented behaviors (61). Over the long-term, from a few years to a lifetime, well-designed work can increase knowledge accumulation in the form of crystallized intelligence and wisdom, and can increase cognitive reserve thus minimizing the impact of cognitive decline (61).

To date, studies investigating the impact of well-designed work on cognitive performance have been conducted in neurotypical adults with cognitive abilities in the normal range (60, 67–73). Their findings, however, raise the important question as to whether well-designed work can also preserve or improve cognitive functioning in individuals with cognitive impairments. Whereas a few studies have assessed the efficacy of vocational training for individuals with cognitive impairments, they have primarily evaluated the impact of vocational training on quality of life measures, adaptive behaviors, and independent living skills (74–76). To our knowledge, no study has assessed the impact of vocational training or well-designed work on cognitive abilities as evaluated by standard neuropsychological tests in individuals with cognitive impairments.

The factories of the AMIPI—Bernard Vendre Foundation in France (known in French as *Usines de Production, d'Apprentissage et d'Insertion*, or Production, Learning and Insertion Factories) are work environments designed with three main goals: (1) to offer individuals with cognitive impairments the opportunity to improve their intellectual, emotional and social well-being; (2) to offer gainful employment to individuals with cognitive impairments; and (3) to produce high quality electrical cables and harnesses for the automobile sector. Specifically, the AMIPI Foundation provides their workers, henceforth referred to as operators, a safe and stable work environment in which to acquire many different skills including but not limited to electrical cable and harness production, reception of orders and stock management, order preparation and dispatch, quality control of components and finished products, team leader assistance, and employee formation. In addition to training them for work at the AMIPI factories, this vocational training prepares operators who are willing and able to transition to the open labor market. Indeed, over the past 50 years more than 1,500 AMIPI operators with recognized cognitive impairment-status have transitioned to permanent jobs in the open labor market (integrated employment). These results suggest that for individuals with cognitive impairments, working at an AMIPI factory may improve cognitive performance, although this has not been tested empirically.

The pedagogical approach employed in the AMIPI factories exemplifies well-designed work theory and allows each operator to learn and progress regardless of their abilities. Upon hiring, each individual is evaluated with respect to several visuospatial and psychomotor abilities, and then assigned a position in the factory that corresponds to their ability and allows for success and progression. Throughout their employment, operators are coached by trainers specialized in teaching adults with cognitive impairments. Trainers and factory supervisors assure that operators are not just trained to do a specific task (e.g., produce a particular harness as part of an assembly line), but rather are familiarized with and often trained on multiple aspects, including preceding and succeeding steps. This provides job complexity and job variety, the former being a characteristic of well-designed work (61) and the latter being a key factor in the Job Characteristics Model (JCM) (65, 77, 78), a foundational model in work design. For example, when training a new procedure to an operator over a multiple-day period (e.g., how to produce a particular harness), the trainer first explains what the operator will be doing, and then explains the importance of each successive step in relation to the entire production process. Throughout training, the trainer shows the operator the different components, names them and describes their function, then asks the operator to point out the different components, name them and describe their function [i.e., the trainer follows the pedagogical methods of Séguin (79, 80)], thus giving meaning to the procedure underlying the product that is being manufactured. This provides what is known as job identity and job significance, two key factors in the JCM (65, 77). Target activities are broken down into simplified steps, and operators are trained only on a limited number of steps at a time until each step is acquired. Workstations are equipped with a wide range of aids (e.g., visual cues) that help operators to learn. Learning and improved performance lead to greater autonomy, another feature key to well-designed work. Operators must also interact with other colleagues on the production line, providing opportunities for building social-emotional competencies, another key feature of well-designed work (61). Operators are explained the importance of identifying and learning from errors, are encouraged to exercise problem-solving skills, and are regularly queried about how to resolve problems, what the implications of such problems are on the larger assembly process, and whom to contact when confronted with a problem they cannot solve, again promoting autonomy and intellectually engaging problem-solving skills. Operators are encouraged, and often required, to become polyvalent and to learn as many different jobs at the factory as possible, again offering job variety. Individual progress sheets or electronic tablets with target production goals are placed at each workstation and allow operators to note and evaluate their performance at regular intervals, providing performance feedback and promoting self-efficacy and autonomy. Operators also receive a report at the end of any specific training that summarizes the points that they have mastered and those requiring further work and training to master, providing further performance feedback from superiors. Finally, the AMIPI Foundation offers continuing education opportunities for their operators targeting job-related knowledge and skills such as information on the automotive sector, quality standards, or product characteristics. Opportunities for personal development are also offered and include, for example, a course on neuroscience and lifelong learning that allows operators to learn about the brain and how to optimize learning, identify transferable skills, and define a long-term professional project for themselves within the Foundation and beyond. These programs provide social support and opportunities for personal development, factors key to well-designed work environments.

**Figure 1:**
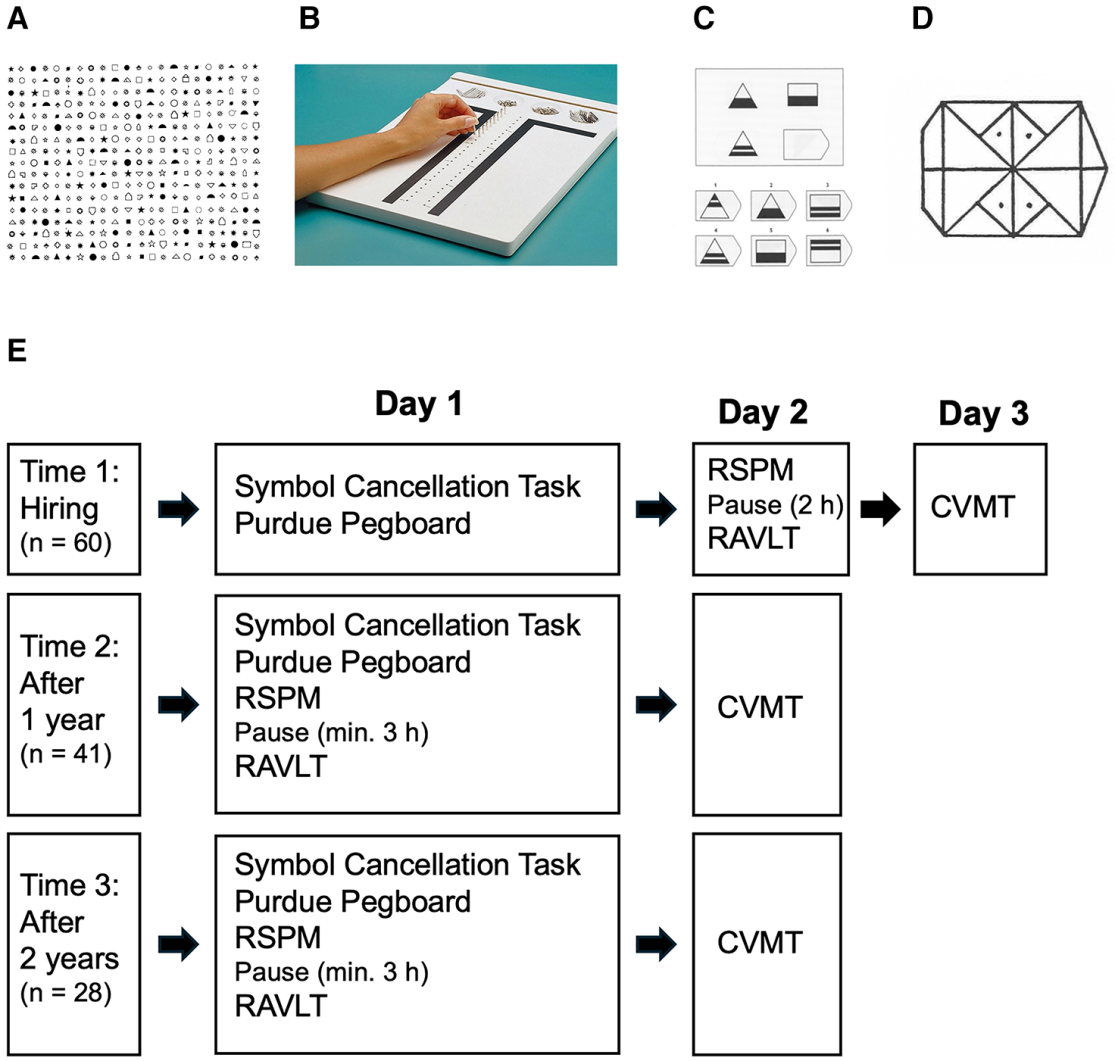
Illustration of four tasks used in the current study. (**A**) Symbol Cancellation Task. (**B**) Purdue Pegboard. (**C**) Raven's Standard Progressive Matrices (RSPM). (**D**) Continuous Visual Memory task (CVMT). NB: Rey's Auditory Verbal Learning task (RAVLT) is not shown (see Table 1 for Structure and Supplementary Tables S2–S4 for word lists). (**E**) Schema of the testing procedure.

The current longitudinal study was designed to assess the potential changes in cognitive capacities exhibited by operators with cognitive impairments during their first two years of working in AMIPI Foundation factories, well-designed work environments. We assessed manual dexterity, bimanual coordination, and procedural learning, sustained and selective attention, verbal recall and recognition memory, visual recognition memory, and abstract reasoning (Figures 1A–D). Improved performance on standard neuropsychological tests across time would suggest that well-designed work environments can improve cognitive functioning in individuals with cognitive impairments, as previously shown for non-impaired individuals (60, 68–71). Below we present the results obtained by operators on these cognitive tests at hiring (Groups 1, 2 & 3), and after working for one year (Groups 2 & 3) and two years (Group 3) in an AMIPI factory.

## 2 Materials and methods

### 2.1 Participants

Participants were 60 French-speaking adults (40 males, *M* age* *= 27.5 years, *SD *= 9.3 years, median = 25.0 years, range = 17–52 years at the time of hiring; 78% right-handed) who were working at the AMIPI—Bernard Vendre Foundation in France. Participants were mostly of European or North and West African descent. Participants were of lower middle-class to middle-class socioeconomic status, consistent with the population of individuals with cognitive impairments in France seeking employment (81).

Participants were recruited if they met at least one of the three following inclusion criteria: (1) They had followed a scholastic path that included specialized educational instruction for individuals with learning difficulties (e.g., ULIS, IME, SEGPA, ITEP, SESSAD, MFR, etc.); (2) They had obtained scores in the “difficult” range on a hiring assessment that evaluated spatial organization and visual memory (e.g., they had difficulties completing these tasks designed to assess individuals with cognitive impairments); (3) They had received handicapped worker status from the French government (i.e., *Reconnaissance de la Qualité de Travailleur Handicapé*, RQTH) due to (a) cerebral trauma (e.g., stroke, cranial trauma, coma, anoxia due to cardiac arrest or other accident); (b) epilepsy; (c) general academic “slowness”, learning and/or memory problems; (d) a diagnosed neurodevelopmental disorder (Trisomy 21, Williams syndrome, etc.), dyslexia, dysgraphia, dyspraxia; (e) an Autism spectrum disorder (ASD; *NB*: individuals with ASD were to be assessed individually by PBL to determine whether they were considered to have cognitive impairments and high-functioning individuals were to be excluded, but we had no self-disclosed ASD individuals that fell into this category); or (f) mental illness (schizophrenia, bipolar disorder, chronic depression, etc.). Supplementary Table S1 reports the number and percentage of individuals who reported to meet each of these inclusion criteria. Exclusion criteria comprised individuals with handicapped worker status due exclusively to a physical handicap, and non-impaired individuals who followed a normal scholastic path (i.e., they did not have RQTH status).

### 2.2 Recruiting procedure

The AMIPI—Bernard Vendre Foundation offers permanent contracts only to individuals with recognized cognitive impairments. Candidates who were offered a permanent contract at AMIPI were assessed by one of the trainers involved in the study (M-LB) to determine if they met the inclusion criteria and were not excludable. Potential participants were explained that the objective of the study was to follow their progression over their first two years of work at the factory, and then explained what the nature of their participation would entail, that they had the right to decline to participate, the right to withdraw from the study at any time, and that their decision would have no impact on their employment, salary, or treatment at the AMIPI factory. They were informed that all testing would take place during normal work hours, and that they would be paid their normal hourly wage during their participation. Prospective participants were given the information and consent form and required to take at least 24 h before deciding to participate. For individuals with a legal guardian, the information and consent forms were transmitted to the legal guardian to confirm their participation and give their authorization. Human subjects research was approved by the Committee for Ethics in Research of the University of Paris—Descartes (CER Paris-Descartes; Paris, France; project no 00012019–20) and was in accordance with the code of ethics of the World Medical Association (Declaration of Helsinki) for experiments involving human subjects in research.

### 2.3 General testing procedures

Participants were tested by trainers working at the AMIPI—Bernard Vendre Foundation. All testing took place from Monday through Friday, from 8:00 am to 6:00 pm in a quiet testing room at the AMIPI factory where the operator was employed. Operators were tested either individually or in groups generally of up to 3 participants at a time for tests that permitted several participants to be tested simultaneously. Participants were seated at a table; obscuring dividers were placed between them during group testing. Testing took a total of approximately 3.5 h at each time point, and operators were tested over three days for testing at hiring (T1), and over two days for testing after working for one year (T2) or two years (T3) in an AMIPI factory (Figure 1E).

### 2.4 The cognitive test battery

We used a battery of five tests to assess a variety of cognitive capacities (Figures 1A–D).

#### 2.4.1 Sustained and selective attention

Whereas numerous attention tasks exist, and are highly normed, most rely on the detection of letters or numbers. Since many AMIPI operators may have reading disabilities, including dyslexia, these types of tasks are inappropriate. We thus assessed sustained attention using the Symbol Cancellation Task (Figure 1A), which requires participants to use a red pen to cross out one type of symbol (e.g., a circle with a horizontal line through it) amongst an array of randomly placed symbols on an A4 piece of paper (210 cm wide by 297 cm long). Participants were given 40 sec to complete the task. We recorded the number of target symbols (max = 59) and the number of incorrect lures (max = 310) that were crossed out in 40 s, and we report the number of targets crossed out per second.

#### 2.4.2 Finger and manual dexterity, bimanual coordination, and procedural learning

The Purdue Pegboard (82, 83) consists of an acrylic board with two parallel rows of 25 holes, in which participants must place cylindrical metal pins (Figure 1B). At the top of the board are four shallow wells in which the different elements (pins, washers, and collars) can be placed. Participants first place pins with their dominant hand for 3 trials of 30 sec each, then with their non-dominant hand for 3 trials of 30 sec each, and then with both hands alternately for 3 trials of 30 sec each. Finally, participants construct assemblies using their left and right hand alternately to place a washer, a pin, a collar, and another washer, in this exact order. Participants have 60 sec to create as many assemblies as possible, and the number of individual elements placed on the Pegboard is recorded. There is an approximately 2 min interval between trials.

#### 2.4.3 Verbal memory

We used the Rey's Audio Verbal Learning Test [RAVLT (84);] to assess verbal memory. Table 1 summarizes the testing paradigm. Participants were tested on a different word list at each time point (T1, T2 and T3; see Supplementary Tables S2–S4). Since we could not be sure that the three lists were of equivalent difficulty, we normalized the performance of AMIPI participants by dividing their performance by the average performance of a group of control individuals on the same word list acquired in the context of a separate study. Control participants were 60 individuals (48 women, *M* = 34.5 years, SD = 10.2 years, median = 35.0 years, range 19–51 years), with 20 participants tested on each list. Control participants were tested online and completed the Raven's Standard Progressive Matrices task during the 30 min interval that separated Trial 7 from the unannounced Trial 8.

**Table 1:** Structure of the Rey's audio verbal learning task (RAVLT).

Trials 1–5: List A read aloud by experimenter and recalled by participant (90 s for recall).
Trial 6: List B read aloud by experimenter and recalled by participant (90 s for recall).
Trial 7: Participant is asked to recall List A (90 s for recall).
Trial 8: Unannounced recall trial after a 30 min delay, participant asked to recall List A (90 s for recall).
Recognition Test: 15 words from List A, 15 words from List B and 20 novel lures read aloud by the experimenter, participant is asked to respond affirmatively to words that they recall being on List A.

#### 2.4.4 Abstract reasoning

Abstract reasoning was assessed using the Raven's Standard Progressive Matrices (RSPM; Figure 1C). The RSPM is a nonverbal, culture-fair test that serves as a proxy for fluid intelligence (85–88). The RSPM, consists of five series of 12 increasingly difficult multiple-choice questions in which the missing element that completes a set of items with a specific pattern of visual geometric designs must be identified. Participants had unlimited time to complete the task.

#### 2.4.5 Visual memory

We evaluated visual memory using the Continuous Visual Memory test [CVMT (89) (Figure 1D)]. The CVMT assesses a participant's ability to detect seven repeated, abstract target symbols whenever they occur amongst a series of 96 intermixed target and lure symbols presented sequentially. While the abstract symbols are being shown one after another, participants must respond to each symbol as “old” (repeated target symbol) or “new” (lure symbol). The CVMT also assesses long-term memory with an unannounced 30 min delayed recognition test, during which each target symbol is presented simultaneously with six other similar-looking symbols from the same family and the participant must indicate which symbol is the target symbol.

### 2.5 Data and statistical analyses

**Figure 2:**
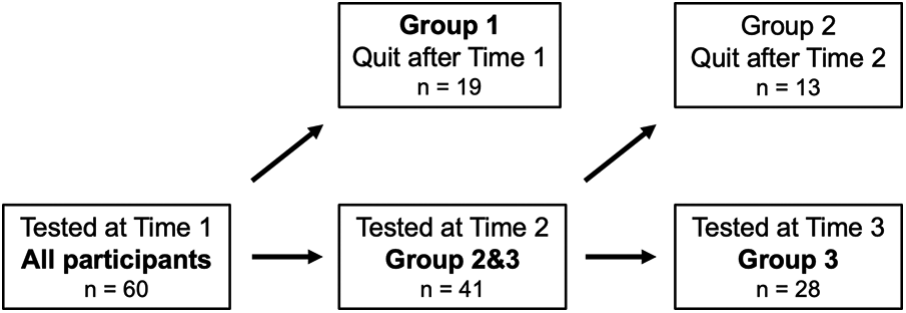
Participants in the study at Time 1 (T1), Time 2 (T2) and Time 3 (T3). Sixty operators were tested at T1. After T1, 19 operators left the AMIPI Foundation and thus were no longer eligible to continue in the study. The remaining forty-one operators were tested again at T2 (referred to as Group 2&3). After T2, 13 more operators left the AMIPI Foundation. The remaining twenty-eight operators were tested again at T3 (referred to as Group 3).

Although this was a longitudinal study, for analysis purposes we created three groups of participants (Figure 2; Table 2). Group 1 consisted of the 19 participants who completed the battery of tests at T1 only and quit working at the AMIPI factories between T1 and T2. Group 2 consisted of 13 individuals who completed the battery of tests at T1 and T2 and quit working at the AMIPI factories between T2 and T3. Group 3 consisted of 28 individuals who completed the battery of tests at T1, T2 and T3. Because Group 2 contained only 13 individuals, statistical analyses including this group alone were not reliable. We thus combined Groups 2 and 3 to create a group of 41 individuals (denoted Group 2&3).

**Table 2:** Demographic information for the participants in each group at T1 (hiring time).

	M/F	Right/Left	Age ± SD (y)	Median age (y)	Min age (y)	Max age (y)
	% Male	% Right				
Group 1	14/5	16/3	29 ± 10	31	17	44
*n* = 19	74%	84%				
Group 2	11/2	10/3	31 ± 10	29	18	52
*n* = 13	85%	77%				
Group 3	15/13	21/7	25 ± 8	21	17	48
*n *= 28	54%	72%				
Group 2&3	26/15	31/10	27 ± 9	23	17	52
*n* = 41	63%	76%				

In the interest of transparency, we compared the results of Group 1 with those of combined Group 2&3 at T1. We thus present three main analyses for each of the five cognitive tests: At T1, we compare the results of Group 1 with those of combined Group 2&3 to determine whether there are any quantitative differences between the individuals in these two groups (i.e., individuals that quit AMIPI before the end of the first year, and those that stay for at least 1 or 2 years). We then compare the results of Group 2&3 at T1 and T2. Finally, we compare the results of Group 3 at T1, T2, and T3. Table 2 presents the demographics of each of the three different groups at T1 (hiring).

Since there was no consistent effect of gender or age (data not shown), data from all participants within a group were combined for analysis and presentation. In the interest of space and legibility, we only summarize the findings in the main text, but have placed all detailed statistical results in the Supplementary Materials (Supplementary Tables S5–S7). We did not use corrections for multiple comparisons for individual *t*-tests because in our study the risk of reporting a difference that may not exist (type I error) is not worse than the risk of missing a difference that may exist (type II error) (90, 91). All statistical analyses were performed with IBM SPSS statistics (version 27.0). Significance level was set at *p* < .05 for all analyses. We reported effect size with partial eta squared (*η*^2^_p_) for ANOVAs, Cohen's *d_s_* for independent samples *t*-tests and Cohen's *d_z_* for paired or one-sample *t*-tests. Data can be requested from the corresponding author.

## 3 Results

### 3.1 Finger and manual dexterity and procedural learning

We evaluated finger and manual dexterity, and procedural learning with the Purdue Pegboard (Figure 1B). First, we averaged the scores of the three trials within each condition (i.e., dominant hand, non-dominant hand and both hands) to compare performance between conditions at each time point. For all groups and at all time points, operators performed better with their dominant hand than with their non-dominant hand, and less well when they had to use both hands alternately (Table 3; all *p* < .01; Supplementary Table S5).

**Table 3:** Purdue Pegboard: mean number and SD (in parentheses) of pins placed for each group and condition at each testing time.

	Dominant hand	Nondominant hand	Alternating hands
**T1:** All operators (*n* = 60)	13.63 (2.33)^a,b^	12.83 (2.82)^a,c^	10.34 (2.12)^b,c^
**T2:** All operators (*n* = 41)	14.15 (2.09)^d,e^	13.18 (1.94)^d^^,f^	10.69 (2.09)^e,f^
**T3:** All operators (*n* = 28)	14.21 (2.43)^g,h^	13.07 (2.10)^g,i^	10.86 (1.89)^h,i^

Superscript letters indicate significant differences (*p* < .05) between the denoted means, whereas superscript numbers indicate a trend (*p* < .1) between the denoted means. For example, for T1, a is different than a, b is different from b, and c is different from c. See Supplementary Table S5 for statistical details.

At T1, between group comparisons revealed that Group 1 did not differ from Group 2&3 on any of the measures (Table 4; Supplementary Table S6). Comparing the performance of Group 2&3 between T1 and T2, a trend was observed for the dominant hand score to improve (*p* = .070), but no differences were observed for the non-dominant hand or alternating hand scores. In contrast, the score for the assemblies was higher at T2. Comparing the performance of Group 3, although no improvements were observed for the dominant hand, non-dominant hand, or alternating hand scores, the assembly score improved from T1 to T2 (*p* = .024) and remained stable between T2 and T3 (*p* = .463). Overall, performance on the assembly task increased 8.5% from T1 to T3 (*p* = .011), suggesting an improvement in procedural memory.

**Table 4:** Purdue Pegboard: mean number and SD (in parentheses) of pins or assembly components placed for each group and at each testing time.

	T1	T2	T3
Group 1			
Dominant hand	14.04 (2.29)		
Non-dominant hand	12.51 (3.76)		
Alternating hands	10.02 (2.71)		
Assemblies	28.21 (7.35)		
Group 2&3			
Dominant hand	13.45 (2.35)^1^	14.15 (2.09)^1^	
Non-dominant hand	12.98 (2.30)	13.18 (1.94)	
Alternating hands	10.48 (1.81)	10.69 (2.09)	
Assemblies	26.90 (6.31)^a^	28.76 (6.79)^a^	
Group 3			
Dominant hand	13.83 (1.95)	14.15 (2.05)	14.21 (2.43)
Non-dominant hand	13.31 (1.9)	13.36 (1.78)	13.07 (2.10)
Alternating hands	10.54 (1.48)	10.88 (2.07)	10.86 (1.89)
Assemblies	27.29 (6.00)^b,c^	29.06 (6.31)^b^	29.61 (6.88)^c^

Superscript letters indicate significant differences (*p* < .05) between the denoted means, whereas superscript numbers indicate a trend (*p* < .1) between the denoted means. See Supplementary Table S6 for statistical details.

### 3.2 Attention

We evaluated sustained and selective attention with the Symbol Cancellation Task (Figure 1A). At T1, Group 1 did not differ from Group 2&3 in the number of targets crossed out per second Table 5; Supplementary Table S6). Comparing the performance of Group 2&3 operators between T1 and T2, the number of targets crossed out per second increased by 22% between time points. The performance of Group 3 operators differed between T1, T2 and T3: It improved from T1 to T2 (*p* = .044) and remained stable between T2 and T3 (*p* = .586). Overall, their performance on the attention task increased 18.3% from T1 to T3 (*p* = .035).

**Table 5:** Symbol cancellation task: mean number and SD (in parentheses) of symbols crossed out per second.

	T1	T2	T3
Group 1	0.81 (0.33)		
Group 2&3	0.72 (0.27)^a^	0.88 (0.30)^a^	
Group 3	0.71 (0.28)^b,c^	0.82 (0.29)^b^	0.84 (0.35)^c^

Superscript letters indicate significant differences (*p* < .05) between the denoted means, whereas superscript numbers indicate a trend (*p* < .1) between the denoted means. See Supplementary Table S6 for statistical details.

### 3.3 Rey's auditory verbal learning task (RAVLT)

We evaluated short-term and long-term verbal memory abilities using the RAVLT. Since this task is often used as an episodic or source memory task requiring individuals to recall the words that they previously heard, we used different lists at different time points. However, since it is difficult to assure that each of the three lists is of equal difficulty, we analyzed the performance of the operators as a ratio of the performance of a group of control participants (described as the proportion of words). We tested 60 control individuals in the same age range, with 20 individuals tested on each list. For the measures of interest, we then divided the individual score of each operator by the average score of the control group for a particular list.

First, we analyzed the proportion of words recalled by operators on the 5th immediate recall trial of the RAVLT (Table 6; Supplementary Table S6): At T1 (hiring), Group 1 did not differ from Group 2&3. We did not find differences in the proportion of words recalled on the 5th immediate recall trial for Group 2&3 between T1 and T2, nor for Group 3 between T1, T2 and T3.

**Table 6:** Rey's auditory verbal learning task: mean proportion and SD (in parentheses) of words recalled on trials 5 and 8 (after a 30 min interval) on the RAVLT.

	T1	T2	T3
Trial 5			
Group 1	0.82 (0.18)		
Group 2&3	0.75 (0.15)	0.78 (0.20)	
Group 3	0.77 (0.15)	0.79 (0.22)	0.77 (0.20)
Trial 8			
Group 1	0.72 (0.24)		
Group 2&3	0.66 (0.23)	0.70 (0.25)	
Group 3	0.70 (0.23)	0.73 (0.25)	0.71 (0.24)

Their were no differences between any of the denoted means. See Supplementary Table S6 for statistical details.

Second, we analyzed the proportion of words recalled by AMIPI operators on the unannounced 8th trial, which followed the 30 min delay (Table 6; Supplementary Table S6): At T1 (hiring), Group 1 did not differ from Group 2&3. For Group 2&3 operators we did not find differences in the proportion of words that they recalled on the 8th trial after a 30 min delay between T1 and T2, nor for Group 3 between T1, T2 and T3.

Finally, we analyzed the results of the delayed recognition test during which participants were presented with 15 words from List A, 15 words from list B (read and recalled once during Trial 6) and 20 new words (lures) in a pseudo-random order, and participants had to respond whether these words were part of list A or not. We used Signal Detection Theory measures to compare performance between groups and at different time points, including the proportion of words from List A identified as being from List A (Hits), the proportion of novel lures identified as being from List A (False Alarms), the signal detection sensitivity measure *d*′ (the standardized difference between the means of the False Alarms and Hits), and the signal detection response bias measure *c* (the tendency to respond positively (“from List A”) or negatively (“not from List A”) across all stimuli). We also considered the proportion of words from List B identified as being from List A (source memory error).

At T1, the proportion of words from List A identified as such (Hits) by AMIPI operators was lower than that of controls (Table 7; Supplementary Table S7). Nevertheless, AMIPI operators recognized 95% of the number of words that controls recognized, with only 6/60 operators exhibiting a score lower than the controls' lowest score, thus suggesting near normal performance. The proportion of novel lures identified as words from List A (False Alarms) by AMIPI operators was higher than that of controls. Nevertheless, only 3/60 operators exhibited a score higher than the controls highest score, thus again suggesting near normal performance. Source memory errors, the proportion of words from List B identified as words from List A, were also more numerous by AMIPI operators than by controls. The sensitivity measure *d*′ exhibited by AMIPI operators was lower than that of controls, signaling poorer discrimination in AMIPI operators. In contrast, the response bias measure *c* of AMIPI operators did not differ from that of controls.

**Table 7:** Rey's auditory verbal learning task: comparison between AMIPI operators and control participants on the RAVLT recognition trial at time 1 (T1).

	AMIPI operators (T1)		Control participants	
	*M (SD)*	Range	*M (SD)*	Range
Proportion Hits (List A words)	0.931 (0.117)^a^	0.400–1.000	0.977 (0.046)^a^	0.800–1.000
Proportion False Alarms (Novel lures)	0.043 (0.052)^b^	0.000–0.250	0.011 (0.023)^b^	0.000–0.100
Proportion Source Errors (List B words)	0.136 (0.218)^c^		0.007 (0.020)^c^	
Sensitivity measure *d*′	3.92 (0.92)^d^		4.65 (0.69)^d^	
Response bias measure c	0.011 (0.541)		0.057 (0.324)	

Superscript letters indicate significant differences (*p* < .05) between the denoted means, whereas superscript numbers indicate a trend (*p* < .1) between the denoted means. See Supplementary Table S7 for statistical details.

At T1, we found no differences between Group 1 and Group 2&3 (Table 8; Supplementary Table S6) in the proportion of List A Hits, novel lure False Alarms, List B Source Memory errors, sensitivity *d*′, or response bias c. Comparing Group 2&3's performance between T1 and T2, we found no differences between time points in the proportion of Hits, False Alarms, Source Memory errors, sensitivity *d*′, or response bias c. Unexpectedly, Group 3 operators had a lower proportion of Hits at T2 than at T1 (*p* = .004) but not as compared to T3 (*p* = .247), and we found no difference between T1 and T3 (*p* = .120). We found no differences between time points in False Alarms or Source Memory errors. There was a higher sensitivity *d*′ at T1, as compared to T2 (*p* = 0.009) and a trend as compared to T3 (*p* = 0.097), but we found no difference between T2 and T3 (*p* = .209). Finally, there was a slight shift in response bias *c* towards more conservative responses (identifying items as new) from T1 to T3 (T1 vs. T2, *p* = 0.079; T2 vs. T3, *p* = 0.527; T1 vs. T3, *p* = 0.044).

**Table 8:** Rey's auditory verbal learning task: mean proportion and SD (in parentheses) of hits, false alarms, source errors, *d*′, and *c*, on the RAVLT recognition trial.

	T1	T2	T3
Group 1			
Hits (List A words)	0.951 (0.085)		
False Alarms (Novel lures)	0.037 (0.040)		
Source Errors (List B words)	0.077 (0.159)		
*d*′ (Sensitivity)	4.07 (1.09)		
*c* (Response bias)	−0.026 (0.359)		
Group 2&3			
Hits (List A words)	0.921 (0.129)	0.899 (0.140)	
False Alarms (Novel lures)	0.046 (0.056)	0.049 (0.068)	
Source Errors (List B words)	0.163 (0.238)	0.142 (0.236)	
*d*′ (Sensitivity)	3.85 (0.84)	3.69 (1.11)	
*c* (Response bias)	0.028 (0.611)	0.120 (0.541)	
Group 3			
Hits (List A words)	0.942 (0.091)^a^	0.883 (0.145)^a^	0.911 (0.112)
False Alarms (Novel lures)	0.048 (0.065)	0.057 (0.078)	0.039 (0.089)
Source Errors (List B words)	0.171 (0.277)	0.155 (0.275)	0.138 (0.275)
*d*′ (Sensitivity)	3.966 (0.795)^b,1^	3.507 (1.122)^b^	3.755 (1.035)^1^
*c* (Response bias)	−0.010 (0.595)^2,e^	0.177 (0.556)^2^	0.244 (0.434)^c^

Superscript letters indicate significant differences (*p* < .05) between the denoted means, whereas superscript numbers indicate a trend (*p* < .1) between the denoted means. See Supplementary Table S6 for statistical details.

In sum, AMIPI operators did not exhibit improvements in declarative verbal memory on either immediate recall (trial 5), delayed recall (trial 8), or delayed recognition memory, despite some inconsistent variations in Hits and sensitivity *d*′ at T2 for operators from Group 3 and an increase from T1 to T3 in their bias to call items new.

### 3.4 Continuous visual memory task (CVMT)

We evaluated short-term and long-term visual memory abilities using the Continuous Visual Memory Task. For the learning phase, we first evaluated the total score, which represents the number of correct responses, that is when operators responded “old” to target symbols and “new” to lure symbols. At T1, Group 1 did not differ from Group 2&3 in Total Score (Table 9; Supplementary Table S6). For Group 2&3, we found no differences in total score between T1 and T2. In contrast, for Group 3, the total score differed between T1, T2 and T3: it improved gradually from T1 to T2 (*p* = .086) and more importantly from T2 to T3 (*p* = .004), leading to an 8.8% improvement in total score from T1 to T3 (*p* = .005).

**Table 9:** Continuous visual memory task: total score, *d*′, and *c*, mean and SD (in parentheses), and delayed recognition memory mean and SD (in parentheses).

	T1	T2	T3
Group 1			
Total Score	71.42 (7.45)		
*d*′ (Sensitivity)	1.92 (0.52)		
*c* (Response bias)	−0.59 (0.40)		
Delayed Recognition	3.47 (2.01)		
Group 2&3			
Total Score	72.02 (6.96)	73.98 (6.48)	
*d*′ (Sensitivity)	1.74 (0.68)^a^	2.06 (0.64)^a^	
*c* (Response bias)	−0.42 (0.48)^b^	−0.57 (0.46)^b^	
Delayed Recognition	3.80 (1.71)^c^	4.41 (1.53)^c^	
Group 3			
Total Score	72.07 (7.01)^1,d^	75.11 (6.23)^1,e^	78.40 (8.24)^d,e^
*d*′ (Sensitivity)	1.83 (0.78)^f,g^	2.19 (0.65)^f,2^	2.42 (0.74)^g,2^
*c* (Response bias)	−0.40 (0.54)	−0.57 (0.49)	−0.50 (0.46)
Delayed Recognition	3.96 (1.82)^h,i^	4.64 (1.42)^h^	5.07 (1.51)^i^

Superscript letters indicate significant differences (*p* < .05) between the denoted means, whereas superscript numbers indicate a trend (*p* < .1) between the denoted means. See Supplementary Table S6 for statistical details.

As for the RAVLT, we compared Signal Detection Theory measures between groups and at different times points (Table 9). For the sensitivity measure *d*′, Group 1 did not differ from Group 2&3 at T1. Comparing the performance of Group 2&3 operators between time points, the sensitivity *d*′ improved between T1 and T2. For Group 3 operators, the sensitivity *d*′ improved between T1, T2 and T3, exhibiting a large increase between T1 and T2 (*p* = .002), and more gradual improvement between T2 and T3 (*p* = .095), leading to a 32% difference in *d*′ from T1 to T3 (*p* < .001).

For the response bias measure c, Group 1 and Group 2&3 did not differ at T1 (Table 9; Supplementary Table S6), and both groups tended to say “old”. For Group 2&3, the response bias *c* became more negative (i.e., higher tendency to say “old”) between T1 and T2. When considered separately, the response bias *c* of Group 3 did not differ between time points. Altogether, the response bias *c* indicated a tendency to consider items as old rather than new, and this tendency was slightly more pronounced at T2 than at T1.

Finally, we evaluated long-term visual recognition memory for abstract symbols after a 30 min delay (Delayed Recognition; Table 9; Supplementary Table S6). At T1, Group 1 and Group 2&3 did not differ in the number of correct responses (i.e., identifying the repeated target symbol). In contrast, the number of target symbols correctly recognized by Group 2&3 increased between T1 and T2. Similarly, the number of target symbols correctly recognized by Group 3 increased from T1 to T3, including a 17% increase from T1 to T2 (*p* = .014), and a more gradual increase from T2 to T3 (*p* = .117), leading to a 28% improvement from T1 to T3 (*p* = .001).

In sum, AMIPI operators exhibited improvements in short-term visual recognition memory between testing times. In immediate recognition tests, the sensitivity measure *d*′ increased between T1 and T2, whereas the total score improved more importantly between T2 and T3. The response bias measure *c* showed some slight variations but remained overall negative, indicating a bias towards responding “old” to stimuli. In the delayed recognition memory test, the total number of repeated symbols correctly recognized by the operators increased between T1 and T2.

### 3.5 Raven's standard progressive matrices (RSPM)

We evaluated the evolution of reasoning and logic abilities in AMIPI operators with the RSPM. Operators had unlimited time to complete the entire series of 60 questions. The average score across all operators and time points was 37 (SD = 11; Median = 39), translating approximately to an IQ of between 83 and 90 depending on the age of the operator (92), situating them in the range of median scores (class 5 of 9) of unqualified French workers in the same age range (93), just below or within the median scores (class 3–5 of 9) of French manual laborers depending on the age of the individual operator (93), and approximately equivalent to the 50th percentile of 8–9.5-year-old children (85, 94). Nevertheless, individual RSPM scores ranged from 12–55, translating to IQ scores lower than the bottom of the normal range (i.e., below 70) to approximately 115, respectively (92). Indeed, across all groups, 14 of the 60 participants (23%) had RSPM scores of 47 or above on at least one test, which is equivalent to having an IQ of 100 or more (92), suggesting that some operators identified as having a cognitive impairment may have cognitive, personality or neurodevelopmental disabilities, but not necessarily below average IQ.

At T1, Group 1 and Group 2&3 did not differ in the total number of correct responses (Table 10; Supplementary Table S6). Similarly, we found no differences between the number of correct responses for Group 2&3 between T1 and T2, or for Group 3 between T1, T2 and T3. In sum, the RSPM score of AMIPI operators did not differ between testing times, indicating that fluid intelligence did not improve.

**Table 10:** Raven's standard progressive matrices: mean, SD (in parentheses), and range of scores.

	T1	Range	T2	Range	T3	Range
Group 1	38.89 (10.30)	15–55				
Group 2&3	36.54 (10.74)	14–54	36.78 (10.54)	15–52		
Group 3	35.59 (11.10)	14–54	35.64 (11.33)	15–52	35.96 (11.06)	12–52

Their were no differences between any of the denoted means. See Supplementary Table S6 for statistical details.

## 4 Discussion

Using a longitudinal study design, we evaluated the impact of well-designed manufacturing work that promotes learning, responsibility, and autonomy, on the cognitive performance of individuals with cognitive impairments. Operators with cognitive impairments who were hired to work at the AMIPI Foundation factories were tested at the time of hiring (T1), one year after hiring (T2), and two years after hiring (T3). We assessed the performance of operators on a battery of neuropsychological tests that evaluated manual dexterity, coordination and procedural learning, attention, recall and recognition verbal memory, recognition visual memory, and fluid intelligence. We found that AMIPI operators exhibited improvements on several measures from T1 to T2 or T3, including procedural memory, attention, and non-declarative visual memory. In contrast, AMIPI operators did not exhibit improvements on overall manual dexterity, declarative verbal memory, or fluid intelligence. We first discuss the results for each of these tasks and the specific cognitive domains evaluated.

### 4.1 Performance on the cognitive battery

#### 4.1.1 Purdue Pegboard

Because the main occupation of operators, especially in their first year of employment at the AMIPI factory, is to manufacture electrical cables and harnesses for automobiles, we hypothesized that manual dexterity and bimanual coordination would improve between testing times. Surprisingly, operators' performance on the Purdue Pegboard did not exhibit systematic improvements when placing single pins with either their dominant or non-dominant hand, nor when placing single pins with both hands in an alternate fashion. Two reasons might explain this result. First, although operators manipulate small components when making cables and harnesses, fine precision grasp requiring high levels of finger dexterity is not usually necessary. Most often the smallest components that operators work with are attached to cables or other larger components, so that they might not work their fine precision grasp sufficiently to exhibit an improvement in the Purdue Pegboard task. Second, it is possible that operators learned the importance of accuracy and quality over speed and quantity, as described by the speed-accuracy trade-off (95). In accordance with the instructions they receive regularly in the factory, when asked to “go as fast as possible”, operators might have understood “go as fast as possible without making ***any*** errors”, inhibiting them from “racing” to place as many pins in the pegboard as possible. However, it is also possible that impairments in the speed-accuracy trade-off, e.g., an inability to intentionally sacrifice accuracy to perform more rapidly, are more generally linked to cognitive impairments. Indeed, studies in individuals with Down syndrome (96), and Attention Deficit/Hyperactivity Disorder (97; but see 98) identified speed-accuracy trade-off impairments in these populations, suggesting that the speed-accuracy trade-off is a cognitive process sensitive to factors underlying neurodevelopmental abnormalities.

In contrast, operators improved on the assembly task that requires bimanual coordination to place a washer, a pin, a collar, and another washer, and which has an important memory component. It is unclear exactly which type of memory participants recruited when learning and then making assemblies, but rapid motor learning of this type has been shown to implicate the motor cortex (99, 100) and the striatum (101, 102). Our results suggest that daily work activity has made operators more performant and able to learn new motor sequences more quickly.

The performance of AMIPI operators can be compared to the established norms for the Purdue Pegboard (83). The original publication included large samples of college men and women, military veterans, and men and women employed in industry. Industrial men consistently scored lowest. For the assembly task, they only performed one 60 sec trial and placed on average 33.1 (SD = 6.3) components, whereas across all three trials AMIPI participants placed on average 27.3 (SD = 6.6) components at T1, and 29.6 (SD = 6.9) components at T3. AMIPI operators thus exhibited performance that was between 83% (T1) and 90% (T3) that of male, American industrial employees in the mid-1940s, placing them around the 25th percentile by T3 (83).

A later study conducted in the mid 70's compared the performance of individuals with cognitive impairments on a standard version of the Purdue Pegboard and an automated version where instructions were presented via videotape (103). Participants attended a vocational training center in New York City and had an average age of 27 years (SD = 7 years) and a mean IQ of 60 (range 45–73). Since the authors were interested in test-retest reliability, they only tested each subtask once at T1 (i.e., 1 × 30 s for dominant hand, 1 × 30 s for non-dominant hand, 1 × 30 s for both hands alternately, and 1 × 60 s for the assemblies), and once again at T2, after a 2-month interval. Since we are specifically interested in the assembly task, we present the results of AMIPI operators only on the first trial (of three) of the assembly task at T1, T2, and T3. Moreover, since Guarnaccia and colleagues considered the automated presentation of the instructions to be more reliable, we compare AMIPI operators with the vocational center participants in the automated task condition. Participants from the vocational training center placed 24.1 (SD = 6.8) components at T1 and 22.7 (SD = 9.0) components at T2, thus failing to exhibit any improvement in performance nor test-retest practice effects over the two-month period. AMIPI operators placed 25.7 (SD = 6.8) components at T1, 27.3 (SD = 6.8) components at T2, and 28.6 (SD = 7.0) components at T3. Accordingly, AMIPI operators exhibited initial performance (i.e., prior to beginning work at AMIPI) that was similar to another group of individuals with cognitive impairments attending a vocational training center, and by the end of their second year of work at AMIPI achieved approximately 90% of the performance of men working in industrial jobs and exhibiting an 11% overall improvement.

#### 4.1.2 Symbol cancellation task

Attentional processes are highly solicited during manufacturing work, so we used a symbol cancellation task to evaluate sustained and selective attention. Operators improved their performance by a total of 18% and crossed out more target symbols after one year and two years of work at AMIPI. Their performance of 0.71 targets/sec at T1 and 0.84 targets/sec at T3 was much better than that of a group of indigenous Amazonians with very little or no education who crossed out on average 0.18 targets/sec on the same cancellation task (104). In contrast, AMIPI operators performed less well than a normative group of healthy, typical 18–50-year-old adults with a minimum of 12 years of education, who crossed out on average 1.06 targets/sec (105). The performance of the AMIPI operators thus ranged from 67% that of typical individuals at T1 to 79% that of the typical individuals at T3.

Nevertheless, it is possible that the attentional processes of AMIPI operators improved more than the 18% that we observed, but that speed-accuracy trade-offs limited their ability to exhibit greater improvements (95). Indeed, operators only crossed out a total of 5 lure symbols in the 129 times that the task was administered, showing that even when instructed to “go as fast as possible” they were concentrated and precise and did not precipitate.

#### 4.1.3 Rey's auditory verbal learning task

Verbal memory is critical to everyday functioning in both professional and personal settings. Because AMIPI trainers understand the importance of accurate technical vocabulary, they are disciplined about using this vocabulary and insist that operators use the proper technical vocabulary when describing materials and procedures. Since verbal memory is a type of semantic memory, which is a type of declarative memory, performance on verbal memory tasks can provide insight into other types of declarative learning and memory. We were thus interested in assessing whether the verbal memory abilities of operators improved across time.

Verbal memory was evaluated with the RAVLT, and we used different word lists for the three test times because we were concerned that participants could remember some words from the lists used the previous years. However, since the equivalence of word lists cannot be guaranteed, we analyzed the performance of AMIPI operators in relation to a group of control participants in the same age range and tested on the same word lists. The performance of AMIPI operators was essentially the same for all measures at each time point, both for short-term memory tested immediately after learning (RAVLT Trial 5) and long-term memory tested after a 30 min delay (RAVLT Trial 8). On short-term memory trials (RAVLT Trial 5), operators recalled on average 77% of the number of words that control participants recalled, whereas on long-term memory trials (RAVLT Trial 8), operators recalled about 70% of the number of words that control participants recalled, thus suggesting relatively greater impairments in long-term verbal memory.

In contrast, operators exhibited near normal recognition memory, with scores between 89% and 96% that of control participants. Importantly, although their sensitivity measure *d*′ was less good than controls, they made very few false alarms (i.e., saying that novel lures came from List A) or source memory errors (saying that words from List B came from List A). Nevertheless, whereas most operators refuted the novel lures as being from List A, a few had higher rates of false alarms. Because some lures were semantically or phonologically related to words from List A and List B, the memory of some AMIPI operators may thus be considered less precise or less specific, or more prone to creating false memories (106). For source memory errors (attributing words from List B to List A), a few operators made many errors. Indeed, 8 of 60 operators identified more than half of the words from List B (i.e., >8 of the 15 words) as being from List A. It is unclear if these were actual source memory failures or if these participants misunderstood the instructions which required them to say “yes” only to those words that were from List A (“the list that was read 5 times”). Nonetheless, it is important to note that they correctly refuted the novel word lures on this same recognition memory task. Only one operator systematically identified novel lures as words from List A, raising the possibility that this participant had more persistent and systematic source memory or attention/comprehension difficulties.

Importantly, the fact that AMIPI operators did not show improvement on the delayed recall test (Trial 8) between T1, T2 and T3 demonstrates that they were not able to explicitly take advantage of knowledge gained from previous experiences with the task: One would expect that knowing that a delayed recall test was part of the testing would help to improve performance at T2 and T3 (107). This was not the case and thus either argues against their ability to benefit from explicit practice effects or, in contrast, suggests that even if they knew it was advantageous, their stable short-term verbal memory span precluded them from holding more words in memory.

In sum, AMIPI operators did not show any improvement in verbal memory with time. Compared to controls, delayed recall memory was more impacted than immediate recall memory, which was more impaired than recognition memory. The fact that their verbal memory did not improve might be explained by the fact that even though AMIPI trainers systematically use proper technical vocabulary and insist that operators also use this vocabulary, this vocabulary is highly specialized. Our results suggest that the ability of AMIPI operators to learn and use a small number of words directly related to their work practice is not correlated with general improvements in short- or long-term verbal memory capacity.

#### 4.1.4 Continuous visual memory task

The CVMT assesses immediate and delayed recognition memory for visually presented abstract symbols. Participants need only respond “old” or “new” based on whether they think that they have already seen the same symbol.

AMIPI operators gradually improved their visual recognition memory abilities as evidenced by several measures. First, their total score for short-term memory during the learning phase increased from T2 to T3. Second, the sensitivity measure *d*′ during the same trials improved from T1 to T2. Third, long-term visual recognition memory (after a 30 min delay) improved from T1 to T2. Based on published norms for the CVMT (89), we determined the percentage of AMIPI operators who performed within the normal range for the total score for short-term (≥74/96 for 18–29-year-olds, ≥71/96 for 30–49-year-olds) or long-term (≥4/7 for 18–29-year-olds, ≥3/7 for 30–49-year-olds) visual recognition memory. For short-term memory, the percentage of operators scoring in the normal range increased from 45% of 60 operators at T1, to 63% of 41 operators at T2, to 79% of 28 operators at T3 [*χ*^2^_(2)_ = 9.528, *p* = .009]. For long-term memory, the percentage of operators scoring in the normal range increased from 62% of 60 operators at T1, to 80% of 41 operators at T2, to 89% of 28 operators at T3 [*χ*^2^_(2)_ = 8.953, *p* = .012]. It is unlikely that mere practice effects or familiarity with the stimuli were responsible for improvements on the CVMT for two reasons: First, because the visual stimuli are complex and abstract, and because the visual stimuli within a family are highly similar, precise long-term visual and verbal encoding is difficult and unlikely. Second, because both target stimuli and lures are seen multiple times throughout the test, first during the learning phase (6 times and 1 time, respectively), second during the test phase after a 30 min delay, and again during a final control visual discrimination test (data not shown), all stimuli would be familiar at retest, not just the target stimuli.

The creators of the CVMT evaluated its construct validity and reported that the sensitivity measure *d*′ is moderately associated with verbal, visual, and attentional factors, whereas the delayed visual recognition memory performance was a factorially “pure” measure of visual memory (108). Germane to the current study, they did not find any correlation between CVMT performance and number of years of education (89) or different levels of scholastic achievement in children of the same age (109). In sum, the CVMT appears to evaluate specifically visual recognition memory and to be relatively free from the influence of either fluid (g*_f_*) or crystallized (g*_c_*) intelligence. From T1 to T3, AMIPI operators exhibited improvements in immediate and delayed visual recognition memory (total score and *d*′), ultimately shifting their total score performance from the 1st percentile to the 16th percentile as compared to normative samples (89).

#### 4.1.5 Raven's standard progressive matrices

The RSPM measures nonverbal abstract reasoning, a proxy for fluid intelligence (g*_f_*) (86, 87, 110). AMIPI operators did not exhibit any improvements on the RSPM across the different time points. It is believed that absolute fluid intelligence increases until early adulthood (15–20 years of age), and then begins to decline, both in individuals with typical as well as below average IQs (111–120). Nevertheless, practice effects have been shown to impact RSPM scores in typical individuals (87, 121), thus making it seem as if IQ does not decrease as much as it actually does when participants are followed longitudinally (121). For example, in one study in which elderly participants took the RSPM several times, with a mean delay of 24 months and a range from several months to more than a decade, the authors reported an average practice effect that conferred a 2-point advantage on the second test, but no further improvements after that (87). In our study, AMIPI operators did not exhibit any improvement in performance across testing times, each one year apart. Because AMIPI operators were relatively young (27.5 ± 9.3 years), age-related cognitive decline should be relatively minor. We can thus assume that either (1) work at the AMIPI factories did not improve fluid intelligence and there was no impact of practice; (2) work at the AMIPI factories slightly improved fluid intelligence, but that this improvement was off-set by slight age-related cognitive decline; or (3) practice did engender minor improvements, but that this improvement was off-set by slight age-related cognitive decline.

#### 4.1.6 Summary

Altogether, our data suggest that AMIPI operators showed improvements in some cognitive domains, including procedural memory, sustained and selective attention, and short-term and long-term visual recognition memory. In contrast, they showed little or no improvement in timed finger dexterity or general bimanual coordination tasks, no improvement in verbal recall or recognition memory, and no improvement in nonverbal abstract reasoning. Interestingly, and perhaps not coincidentally, the tasks on which AMIPI operators exhibited improvements, the Purdue Pegboard assembly task, the attention task, and the visual memory task, are all thought to evaluate nondeclarative, hippocampus-independent processes (101, 122–125). In contrast, two of the three tasks on which AMIPI operators did not exhibit improvements, the verbal recall memory task and the abstract reasoning task, are thought to evaluate declarative, hippocampal-dependent processes (126–132). These findings suggest that certain cognitive domains are susceptible to improvement and far-transfer effects, whereas others are not. Moreover, our findings suggest that susceptible domains can be improved by well-designed work serving as a cognitive training intervention for individuals with cognitive impairments.

### 4.2 Cognitive training in individuals with cognitive impairments

Over the past 20 years the scientific community has taken a large interest in cognitive training, often trying to demonstrate the efficacity and utility of specific cognitive tasks to improve overall cognitive functions. The promise of cognitive training is that the brain is plastic, and that this plasticity is not limited to simple associative or motor learning, such as learning to associate a new word with a new object (e.g., a connector box) or learning to produce a new action (e.g., insert this cable into this connector box), but that this plasticity extends to higher order cognitive functions such as attention, perception, memory and intelligence. However, there is still considerable debate about whether cognitive training is effective, to what degree cognitive training can produce practically significant and individually relevant results, and whether cognitive training is task-specific, or if its benefits can transfer to closely related domains and material (i.e., near transfer), or unrelated domains and material (i.e., far transfer). Many studies have reported that both domain-specific and domain-general cognitive training can result in enhancements in cognitive functions, including executive function, working memory, attention, visual processing and task switching (133–146). In contrast, other studies have either found no impact of cognitive training on long-term cognitive functions, or warned of potential confounds in the evaluation of the impact of cognitive training, including the lack of appropriate control groups and placebo effects (133, 147–158).

The present study was designed to assess the possible impact that well-designed manufacturing work may have on the cognitive abilities of individuals with cognitive impairments and not as a controlled, experimental intervention study. We nonetheless recognize the parallels and thus discuss our findings in relation to other studies that have investigated cognitive training in individuals with cognitive impairments.

Although relatively few, the majority of studies assessing cognitive training in adolescents and adults with cognitive impairments have used computerized “brain-game” interventions, combined with pre- and post-training psychometric evaluations. Researchers have implemented interventions using their own cognitive training programs (159), or commercially available training programs such as Cogmed (160), Scientific Brain Training Pro™ (161), CogniFit^©^ (162), or Guttman NeuroPersonalTrainer® (163). Most studies have used standard neuropsychological tests and behavioral inventories that assess short-term or working memory for verbal and visuospatial information (e.g., digit span, word span, and block span, forward and backward), executive functions such as planning, inhibition and task-switching (e.g., Tower of London, Stroop and set-shifting tasks), and fluid intelligence (e.g., matrix tasks like the Raven's SPM) (159–161, 163). Other studies used the cognitive evaluations that are included with the commercially available training program (162), which are described as qualitatively different from the training exercises. In all these studies, adolescents and adults with cognitive impairments showed improvements in some of the various cognitive capacities following training (*NB*: the studies of McGlinchey et al., 2019, and Siberski et al., 2015, used stringent statistical adjustments for multiple comparisons, which we argue are not appropriate for these kinds of actual observations (see Statistical Analyses Section of the Methods); We thus include their results that do not incorporate adjustments for multiple comparisons for the purposes of this summary statement.) Two of the studies showed what could be considered to be far-transfer effects: McGlinchey and colleagues found that adult individuals with Down syndrome who trained on a series of executive function tasks scored better on a limited number of caregiver-rated measures of everyday executive function after training [if adjustments for multiple comparisons are ignored (161)]. Similarly, Calub and colleagues found that after Cogmed training, children and adolescents with autism spectrum disorder (ASD) or comorbid ASD and Fragile X syndrome scored better on two behavioral inventories assessing on-task behaviors (being able to focus on the task at hand) and repetitive, ritualistic, and pragmatic problems (160).

Germane to the present study, only two of these previous studies assessed fluid intelligence and they found conflicting results. Whereas Van der Molen and colleagues did not find improvements in a group of 95 adolescents (13–16-years-old) with mild to borderline cognitive impairments in Raven's SPM performance following cognitive training with their non-commercial program (159), García-Alba and colleagues found a difference in performance on the Matrix subtask of the KBIT-2 (164; similar to the Raven's SPM) in a group of 10 men (M = 52.7 years) following training with the Guttman NeuroPersonalTrainer® (163). Because this latter study only found improvements in fluid intelligence but not in any other cognitive measure, because its sample size is quite small, and because data on the wait-group performance after their presumed intervention is not shown, its results should be considered with caution.

In contrast, to our knowledge, no studies have evaluated the impact of vocational training or work itself on cognitive performance in individuals with cognitive impairments, which was thus the impetus for the current exploratory study. Indeed, most studies assessing the efficacy of vocational training in individuals with cognitive impairments focused on measures of quality of life, adaptive behaviors, and independent living skills (74–76). For example, one study described the effects of a non-specific, approximately 10-month long vocational training program on the adaptive behavior of 43 young adults with cognitive impairments (18–28-yrs (75)). The vocational training program focused on general adaptive behaviors, such as hygiene, appropriate clothing, responsibility, establishing a time schedule that is appropriate for the activities that need done, and the development of adaptive work skills. Participants' adaptive behavior before and after the intervention was evaluated via the Supports Intensity Scale (SIS; American Association on Intellectual and Developmental Disabilities) on five different scales assessing Home Living, Community Living, Life-long Learning, Employment, and Health and Safety, and thus is an indirect measure of functionality. Participants' scores improved on all scales, from 14.5%–53%. Nonetheless, it is of note that the smallest amount of improvement, 14.5%, was found on the Life-long Learning scale, and the authors remarked that they found no noticeable improvement in more abstract cognitive processes such as literacy or numerical processing (75).

In sum, whereas “brain game” interventions appear to improve intellectual functioning in adolescents and adults with cognitive impairments, showing that these individuals are capable of intellectual improvements even in adulthood, their far-transfer may be somewhat limited. Whereas standard vocational training targeting adaptive skills that are focused on the work environment has many positive impacts on everyday life and thus real-world validity, its impact on cognitive functioning may be limited. In contrast, our study shows that well-designed work can engender cognitive improvements as evaluated by far-transfer tasks in individuals with cognitive impairments. Moreover, these effects are likely to persist as long as the individual continues working, and may even serve to increase cognitive reserve, by increasing the baseline cognitive functioning of the individual from which inevitable age-related cognitive decline would begin (33). As such, our results suggest that well-designed manufacturing work can itself serve as a cognitive intervention. Rather than providing cognitively impaired individuals with a computerized intervention for them to use on their own or at the prompting of a caregiver or educator for a limited period of time, our study shows that well-designed work can provide the intellectual stimulation necessary to improve cognitive abilities in these individuals. Whereas some individuals with more moderate to severe cognitive impairments might never be able to integrate into open market employment, other individuals with milder cognitive impairments will likely be able to improve their cognitive functioning enough to be able to find jobs in the open market. And, in the meantime, they will have acquired valuable social and vocational knowledge and skills to accelerate their integration. However, even for those that cannot integrate, sheltered employment can still provide intellectual, physical and social stimulation that improves overall social and financial well-being and mitigates age-related physical and cognitive decline (33), benefiting the individual, their families and society (165).

## 5 Conclusions

To our knowledge, this is the first study to assess the cognitive benefits of vocational training in individuals with cognitive impairments using neuropsychological assessments. We found that well-designed training and work in a production factory improved the procedural memory, attentional abilities, and visual memory abilities of workers with cognitive impairments. In contrast, we did not observe improvements in finger and manual dexterity or bi-manual coordination, verbal memory, or fluid intelligence. Although our study was limited in the number of cognitive domains that were evaluated, it is suggestive that other intellectual capacities can be improved by ethologically valid and intellectually stimulating well-designed work opportunities for individuals with cognitive impairments.

## Data Availability

The raw data supporting the conclusions of this article will be made available by the authors, without undue reservation.
