# Supplementary material for: Well-designed manufacturing work improves some cognitive abilities in individuals with cognitive impairments
Source: Front Rehabil Sci. 2024 May 14;5:1377133. doi: 10.3389/fresc.2024.1377133 (PMC11135131; doi:10.3389/fresc.2024.1377133)
Supplement: Supplementary file 1 [file Table1.pdf]

Supplementary Table S1. Number and percentage (in parentheses) of operators who self-reported to meet the specific inclusion criteria. RQTH: Handicapped worker status in France (*Reconnaissance de la Qualité de Travailleur Handicapé*). Note: RQTH-status is determined by the state. *The hiring assessment evaluated visuo-constructive abilities, visual memory, bimanual coordination, and manual dexterity. Some workers met two or more inclusion criteria, such as Specialized education and Scores in the "difficult" range on the hiring assessment.

Inclusion Criteria	T1 (n = 60)	T2 (n = 41)	T3 (n = 28)
Specialized education (ULIS, IME, SEGPA, ITEP, SESSAD, MFR, etc.)	48 (80%)	36 (88%)	25 (89%)
Scores in the "difficult" range on a hiring assessment*	25 (42%)	20 (49%)	12 (43%)
RQTH Learning disability, Neurodevelopmental syndrome	7 (12%)	7 (17%)	4 (14%)
RQTH Cranial trauma, stroke, cardiac incident	5 (8%)	3 (7%)	2 (7%)
RQTH Epilepsy	5 (8%)	3 (7%)	2 (7%)
RQTH Mental illness	4 (7%)	1 (2%)	1 (3%)
RQTH Autism	1 (2%)	1 (2%)	1 (3%)

Supplementary Table S2.

Word List 1: Rey's Auditory Verbal Learning Task (RAVLT) French word / English word

List A: (read in this order and then attempted to be recalled)	List B (read in this order and then attempted to be recalled)	Recognition List (50 words) read in this order one time	
Tambour / Drum	Pupitre / Desk	Ceinture / Belt	Fusil / Gun
Rideau / Curtain	Berger / Shepherd	Fenêtre / Window	Teint / Color
Ceinture / Belt	Moineau / Sparrow	Casquette /Cap	Église / Church
Café / Coffee	Soulier / Shoe	Ferme / Farm	Dindon / Turkey
École / School	Fourneau / Oven	Berger /Shepherd	Fontaine / Fountain
Parent / Parent	Montagne / Mountain	Moustache / Mustache	Bateau / Boat
Soleil / Sun	Lunettes / Glasses	Poêle / Stove	Verger / Orchard
Jardin / Garden	Éponge / Sponge	École / School	Parent / Parent
Casquette / Cap	Image / Picture	Cascade / Waterfall	Fleuve / River
Paysan / Farmer	Bateau / Boat	Crayon / Pencil	Paysan / Farmer
Moustache / Mustache	Mouton / Sheep	Habitation / Dwelling	Rose / Rose
Dindon / Turkey	Fusil / Gun	Poisson / Fish	Image / Picture
Couleur / Color	Crayon / Pencil	Soleil / Sun	Maison / House
Maison / House	Église / Church	Moustique / Mosquito	Parrain / Godfather
Rivière / River	Poisson / Fish	Groseille / Currant	Jardin / Garden
		Moineau / Sparrow	Lunettes / Glasses
		Montagne / Mountain	Chaussette / Sock
		Café / Coffee	Soulier / Shoe
		Maçon / Mason	Maîtresse / Teacher
		Rivière / River	Fourneau / Furnace
		Éponge / Sponge	Nid / Nest
		Rideau / Curtain	Enfant / Child
		Pantalon / Pant	Tambour / Drum
		Couleur / Color	Peinture / Painting
		Pupitre / Desk	Mouton / Sheep

Supplementary Table S3.

Word List 2: Rey's Auditory Verbal Learning Task (RAVLT) French word / English word

List A: (read in this order and then attempted to be recalled)	List B (read in this order and then attempted to be recalled)	Recognition List (50 words) read in this order one time	
Livre / Book	Bol / Bowl	Prairie / Meadow	Veste / Jacket
Fleur / Flower	Matin / Morning	Cargaison / Cargo	Cuillère / Spoon
Train / Train	Juge / Judge	Bûche / Log	Piscine / Pool
Tapis / Carpet	Bourse / Purse	Arbre / Tree	Clé / Key
Prairie / Meadow	Insecte / Insect	Bouteille / Bottle	Luge / Sled
Harpe / Harp	Avion / Plane	Doigt / Finger	Avion / Plane
Sel / Salt	Ville / City	Voyage / Trip	Gomme / Eraser
Doigt / Finger	Piscine / Pool	Tapis / Carpet	Sel / Salt
Pomme / Apple	Graine / Grain	Carpe / Carp	Alliance / Ring
Cheminée / Chimney	Brebis / Ewe	Insecte / Insect	Bouton / Button
Bouton / Button	Repas / Meal	Poêle / Stove	Campagne / Countryside
Bûche / Log	Veste / Jacket	Ville / City	Bourse / Purse
Clé / Key	Bouteille / Bottle	Or / Gold	Fleur / Flower
Ballon / Ball	Pêche / Fishing	Beurre / Butter	Bêche / Spade
Or / Gold	Chaise / Chair	Fée / Fairy	Harpe / Harp
		Chaise / Chair	Matin / Morning
		Brebis / Ewe	Ferme / Farm
		Livre / Book	Graine / Grain
		Toit / Roof	Scie / Saw
		Cheminée / Chimney	Repas / Meal
		Juge / Judge	Sol / Floor
		Train / Train	Orteil / Toe
		Poivre / Pepper	Pomme / Apple
		Ballon / Ball	Capuche / Hood
		Bol / Bowl	Pêche / Fishing

Supplementary Table S4.

Word List 3: Rey's Auditory Verbal Learning Task (RAVLT) French word / English word

List A: (read in this order and then attempted to be recalled)	List B (read in this order and then attempted to be recalled)	Recognition List (50 words) read in this order one time	
Poupée / Doll	Assiette / Plate	Clou / Nail	Tarte / Pie
Miroir / Mirror	Bouffon / Buffoon	Sable / Sand	Bois / Wood
Clou / Nail	Colline / Hill	Lit / Bed	Vélo / Bike
Marin / Sailor	Gant / Glove	Poney / Pony	Moto / Motorcycle
Cœur / Heart	Outil / Tool	Bouffon / Buffoon	Tablier / Apron
Désert / Desert	Forêt / Forest	Lait / Milk	Pied / Food
Visage / Face	Eau / Water	Plat / Dish	Scie / Saw
Lettre / Letter	Échelle / Ladder	Cœur / Heart	Désert / Desert
Lit / Bed	Fille / Girl	Souper / Supper	Chemin / Road
Machine / Machine	Pied / Food	Souris / Mouse	Machine / Machine
Lait / Milk	Bouclier / Shield	Enveloppe / Envelope	Bébé / Baby
Moto / Motorcycle	Tarte / Pie	Voiture / Car	Fille / Girl
Musique / Music	Souris /Mouse	Visage / Face	Cheval / Horse
Cheval / Horse	Vélo / Bike	Croûte / Crust	Suie / Soot
Route / Road	Voiture / Car	Terroir / Land	Lettre / Letter
		Colline / Hill	Eau / Water
		Forêt / Forest	Joker / Joker
		Marin / Sailor	Gant / Glove
		Sou / Coin	Capitaine / Captain
		Route / Road	Outil / Tool
		Échelle / Ladder	Facteur / Postman
		Miroir / Mirror	Chanson / Song
		Figure / Face	Poupée / Doll
		Musique / Music	Écurie / Stable
		Assiette / Plate	Bouclier / Shield

Statistical analyses

Since there was no consistent effect of gender or age (data not shown), data from all participants within a group were combined for analysis and presentation. We did not use corrections for multiple comparisons for individual t-tests because in our study the risk of reporting a difference that may not exist (type I error) is not worse than the risk of missing a difference that may exist (type II error). Accordingly, we followed the recommendations of Rothman (1990), who argued that "not making adjustments for multiple comparisons is preferable because it will lead to fewer errors of interpretation when the data under evaluation are not random numbers but actual observations on nature," and Saville (1990), who also argued that a procedure without correction is preferable because it provides greater consistency to compare results between studies. Although some comparisons might be more critical than others, considering all comparisons is essential to characterize the behavior of participants. All statistical analyses were performed with IBM SPSS statistics (version 27.0). Significance level was set at $p < .05$ for all analyses. We reported effect size with partial eta squared (η^2_p) for ANOVAs, Cohen's d_s for independent samples t-tests and Cohen's d_z for paired or one-sample t-tests. Data can be requested from the corresponding author.

Supplementary Table S5. Statistical results for the Purdue Pegboard performance of all operators when placing single pins with the dominant hand, the non-dominant hand, or both hands in alternation, at T1, T2, and T3.

Dexterity (Purdue Pegboard)		Dominant vs nondominant	Dominant vs both alternating	Non-dominant vs both alternating
T1 all operators (n = 60)	$F_{(2,118)} = 80.247$, $p < .001$, $\eta^2_p = 0.576$	$p = .006$	$p < .001$	$p < .001$
T2 all operators (n = 41)	$F_{(2,80)} = 202.992$, $p < .001$, $\eta^2_p = 0.835$	$p < .001$	$p < .001$	$p < .001$
T3 all operators (n = 28)	$F_{(2,54)} = 94.755$, $p < .001$, $\eta^2_p = 0.778$	$p < .001$	$p < .001$	$p < .001$

Supplementary Table S6. Statistical findings for results described in the manuscript text, for Group 1 vs Group 2&3 comparison at T1, for Group 2&3 at T1 vs T2, and for Group 3 at T1 vs T2 vs T3.

	Group 1 vs Group 2&3 (T1)	Group 2&3 (T1 vs T2)	Group 3 (T1 vs T2 vs T3)	Group 3 Post hoc p-values: T1 vs T2	T2 vs T3	T1 vs T3
Dexterity & Procedural memory (Purdue Pegboard)						
Dominant hand	$t_{(58)} = 0.908, p = .368, d_s = 0.252$	$t_{(40)} = 1.861, p = .070, d_z = 0.291$	$F_{(2,54)} = 0.570, p = .569, \eta^2_p = 0.021$			
Non-dominant hand	$t_{(58)} = 0.594, p = .555, d_s = 0.165$	$t_{(40)} = 0.756, p = .454, d_z = 0.118$	$F_{(2,54)} = 0.530, p = .592, \eta^2_p = 0.019$			
Alternating hands	$t_{(58)} = 0.788, p = .434, d_s = 0.219$	$t_{(40)} = 0.648, p = .521, d_z = 0.101$	$F_{(2,54)} = 1.032, p = .363, \eta^2_p = 0.037$			
Assemblies	$t_{(58)} = 0.709, p = .481, d_s = 0.197$	$t_{(40)} = 2.423, p = .020, d_z = 0.378$	$F_{(2,54)} = 4.858, p = .011, \eta^2_p = 0.152$	.024	.463	.011
Attention (Symbol cancellation task)						
Number of symbols	$t_{(58)} = 1.180, p = .243, d_s = 0.327$	$t_{(40)} = 3.038, p = .004, d_z = 0.474$	$F_{(2,54)} = 3.757, p = .030, \eta^2_p = 0.122$	.044	.586	.035
Verbal memory (RAVLT)						
Trial 5 recall	$t_{(58)} = 1.555, p = .125, d_s = 0.432$	$t_{(40)} = 1.211, p = .233, d_z = 0.189$	$F_{(2,54)} = 0.295, p = .746, \eta^2_p = 0.011$			
Trial 8 recall	$t_{(58)} = 1.025, p = .310, d_s = 0.284$	$t_{(40)} = 1.154, p = .255, d_z = 0.180$	$F_{(2,54)} = 0.219, p = .804, \eta^2_p = 0.008$			
Hits (List A)	$t_{(58)} = 0.910, p = .367, d_s = 0.52$	$t_{(40)} = 0.885, p = .381, d_z = 0.138$	$F_{(2,54)} = 4.036, p = .023, \eta^2_p = 0.130$	.004	.247	.12
False alarms (Novel lures)	$t_{(58)} = 0.660, p = .512, d_s = 0.183$	$t_{(40)} = 0.213, p = .832, d_z = 0.033$	$F_{(2,54)} = 0.531, p = .591, \eta^2_p = 0.019$			
Source errors (List B)	$t_{(58)} = 1.421, p = .161, d_s = 0.389$	$t_{(40)} = 0.485, p = .630, d_z = 0.076$	$F_{(2,54)} = 0.187, p = .830, \eta^2_p = 0.007$			
d' sensitivity	$t_{(58)} = 0.871, p = .387, d_s = 0.242$	$t_{(40)} = 0.899, p = .374, d_z = 0.140$	$F_{(2,54)} = 4.135, p = .021, \eta^2_p = 0.133$	.009	.209	.097
c response bias	$t_{(58)} = 0.358, p = .721, d_s = 0.099$	$t_{(40)} = 0.908, p = .369, d_z = 0.142$	$F_{(2,54)} = 2.893, p = .064, \eta^2_p = 0.097$	.079	.527	.044
Visual memory (CVMT)						
Total score	$t_{(58)} = 0.306, p = .761, d_s = 0.085$	$t_{(40)} = 1.388, p = .173, d_z = 0.217$	$F_{(2,54)} = 7.153, p = .002, \eta^2_p = 0.209$	.086	.004	.005
d' sensitivity	$t_{(58)} = 1.038, p = .303, d_s = 0.288$	$t_{(40)} = 3.567, p < .001, d_z = 0.557$	$F_{(2,54)} = 11.125, p < .001, \eta^2_p = 0.292$	.002	.092	<.001
c response bias	$t_{(58)} = 1.318, p = .193, d_s = 0.366$	$t_{(40)} = 2.579, p = .014, d_z = 0.403$	$F_{(2,54)} = 2.295, p = .111, \eta^2_p = 0.078$			
Delayed recognition	$t_{(58)} = 0.661, p = .511, d_s = 0.183$	$t_{(40)} = 2.875, p = .006, d_z = 0.449$	$F_{(2,54)} = 8.236, p < .001, \eta^2_p = 0.234$	.014	.117	.001
Abstract reasoning (Raven's SPM)						
Total score	$t_{(58)} = 0.801, p = .426, d_s = 0.222$	$t_{(40)} = 0.327, p = .746, d_z = 0.051$	$F_{(2,54)} = 0.240, p = .787, \eta^2_p = 0.009$			

Supplementary Table S7. Statistical results for control participants vs AMIPI Operators on RAVLT measures at T1.

Verbal memory (RAVLT)	T1 Controls vs AMIPI Operators
Hits (List A)	$t_{(118)} = 2.825$, $p = .006$, $d_s = 0.516$
False alarms (Novel lures)	$t_{(118)} = 4.462$, $p < .001$, $d_s = 0.815$
Source errors (List B)	$t_{(118)} = 4.550$, $p < .001$, $d_s = 0.831$
d' sensitivity	$t_{(118)} = 4.942$, $p < .001$, $d_s = 0.902$
c response bias	$t_{(118)} = 0.564$, $p = .574$, $d_s = 0.103$
